# The Effectiveness of Salivary Sampling for the Detection and Quantification of *Aggregatibacter actinomycetemcomitans* in Periodontitis Patients

**DOI:** 10.3390/pathogens13121073

**Published:** 2024-12-07

**Authors:** Nabil Khzam, Omar Kujan, Dorte Haubek, Aysen Arslan, Anders Johansson, Jan Oscarsson, Zeinab Razooqi, Leticia Algarves Miranda

**Affiliations:** 1 Dental School, The University of Western Australia, Nedlands, WA 6009, Australia; omar.kujan@uwa.edu.au (O.K.); leticia.algarvesmiranda@uwa.edu.au (L.A.M.); 2 NK Periodontics, Specialist Periodontal Private Practice, Applecross, WA 6152, Australia; 3 Jammerbugt Municipal Dental Service, Skolevej 1, DK-9460 Brovst, Denmark; dorte.haubek@outlook.dk; 4 Department of Odontology, Umeå University, 901 87 Umeå, Sweden; aysenarslan2003282@gmail.com (A.A.); jan.oscarsson@umu.se (J.O.); zeinab.razooqi@umu.se (Z.R.)

**Keywords:** *A. actinomycetemcomitans*, periodontitis, plaque, saliva, cheek swab, qPCR

## Abstract

The objective was to evaluate using unstimulated saliva in detecting *Aggregatibacter actinomycetemcomitans* and to compare the saliva and subgingival and mucosa membrane occurrence of this periodontal pathogen in patients diagnosed with advanced periodontitis. Patients with advanced forms of periodontitis (*n* = 220; mean age: 54.03 ± 03 years) at stage III/IV were sampled. Unstimulated saliva, buccal cheek mucosa, and pooled subgingival plaque samples were collected. The identification of *A. actinomycetemcomitans* was performed using qPCR. A descriptive analysis and Wilcoxon test and analysis of variance were performed. *A. actinomycetemcomitans* was isolated from 28.18% of the subjects. A total of 660 samples were obtained, 220 from unstimulated saliva, 220 from buccal cheek mucosa surfaces, and 220 from pooled subgingival plaque samples. *A. actinomycetemcomitans* was isolated from 21.80% of unstimulated saliva, 19.50% of buccal cheek swabs, and 17.70% of subgingival samples. There was a statistically significant difference between the presence of *A. actinomycetemcomitans* in the unstimulated saliva samples and in the buccal cheek mucosa swab samples and pooled subgingival plaque samples (*p* < 0.001). These results suggest that in advanced periodontitis, unstimulated saliva is representative of pooled subgingival plaque/buccal cheek mucosa samples and its use is adequate in the oral detection of *A. actinomycetemcomitans* in a cohort of patients with stage III and IV periodontitis.

## 1. Introduction

Periodontitis is a common multifactorial oral disease in which oral bacteria play an important role in initiation and progression. The oral cavity provides an environment for more than 700 bacterial species, which is only a small part of those known to be periodontal pathogens, causing inflammation in the tooth-supporting apparatus in susceptible individuals [1]. Periodontitis is linked to other chronic inflammatory disorders including, cardiovascular disease, rheumatoid arthritis, and cancer [2,3]. A triadic group of oral anaerobic bacteria designated the “red complex”, comprising *Porphyromonas gingivalis*, *Treponema denticola*, and *Tannerella forsythia*, has traditionally been categorized as the causative agents of periodontitis [4]. A paradigm shift based on the current model of polymicrobial synergy and dysbiosis suggests that the progression of periodontitis is induced by a more comprehensive dysbiotic microbial community rather than by the selected number of periodontal pathogens [5]. Advances in technology have redefined the oral microbiome into a synergistic and dysbiotic microbial community, rather than microbes belonging to the red complex. The development of novel culture-independent techniques, such as PCR and next-generation sequencing, has identified several previously underappreciated bacteria including the gram-positives *Filifactor alocis*, *Peptostreptococcus stomatis*, and other species [6]. *A. actinomycetemcomitans* is a facultative anaerobic, gram-negative, dysbiotic bacterium that resides in the oral cavity and is a major etiological agent of periodontitis [7]. *A. actinomycetemcomitans* possess a wide range of molecules responsible for causing host immunity dysregulation, including leukotoxin (LtxA) and cytolethal distending toxin (CDT) [8]. The JP2 genotype, which belongs to serotype b of *A. actinomycetemcomitans,* is clinically relevant in the risk of periodontitis, especially in young patients of African descent [9]. *A. actinomycetemcomitans* is an early colonizer and is present before teeth eruption in infants [10]. Once the bacterium colonizes a site, it remains detected for a long time despite periodontal treatment in those subjects with periodontitis. The bacterium is a very stable colonizer that can stay up to 12 years [11,12].

A recent publication revealed that *A. actinomycetemcomitans* used as an intra-nasal vaccine may be effective in preventing the colonization of periodontopathic bacteria in the oral cavity and extra-oral organs [13]. *A. actinomycetemcomitans* is one of seven gram-negative bacteria that can be used in the diagnosis and monitoring of periodontitis [14]. The gold standard of periodontitis diagnosis is based largely on periodontal examination, patient history, and radiographic imaging [15,16]. However, these methods are invasive, time-consuming, and costly and may risk missing the diagnosis of emerging periodontitis. Therefore, using a saliva sample can help in the operation of a rapid, molecular-based, and non-invasive platform (OralDisk) for detecting periodontal pathogens [17].

As the role of *A. actinomycetemcomitans* in future vaccines and molecular diagnostics widens, it becomes very important to evaluate the effectiveness of saliva in the oral detection of this periodontal pathogen. Therefore, the objective of this study was to first evaluate the effectiveness of unstimulated saliva in the oral detection of *A. actinomycetemcomitans* and, secondly, to compare saliva presence to the subgingival and extra-crevicular occurrence of this periodontal pathogen in patients with an advanced form of periodontitis.

## 2. Materials and Methods

### 2.1. Research Subjects

A total of 220 consecutive patients with periodontitis, aged between 18 and 86 years old, were recruited to two private periodontal specialist practices in Perth city, Western Australia. The study was taken place between June 2022 and June 2024. For more information regarding the recruitment process, please refer to the previously published study [18]. To be included in this study, the participants had to be from Western Australia, be 18 years and older, and not be taking any medication known to affect the periodontal tissue (antibiotics, host modulation agents, and painkillers). Participants were excluded from the study if they had any of the following within three months’ time of starting this study: teeth scaling, course of antibiotics, periodontal treatment, and acute periodontal infection.

### 2.2. Clinical Examination

Periodontal, radiographic, and photographic examinations were carried out by one experienced periodontist (NK). In each patient, the examination included measurement and scoring of series of different parameters characterizing periodontitis. For more information regarding the recruitment process, please refer to the previously published study [18].

### 2.3. Case Definition of Periodontitis

Cases with periodontitis were defined according to the 2018 classification of periodontal and peri-implant diseases and conditions [18].

### 2.4. Samples Collection

Samples of unstimulated saliva, cheek swabs, and subgingival plaque samples were collected from each subject. Subjects were asked to refrain from eating and drinking one hour prior to saliva collection. About 2 mL of expectorated whole saliva was collected from each subject (220 samples). In each patient’s subgingival plaque samples were collected. The pooled subgingival plaque samples were collected using sterile universal mini-curette after air drying the area carefully and removing any food debris. The subgingival plaque samples were collected from the most apical portion of the accessible pocket depth. For each patient, subgingival plaque samples were collected from four diseased and four healthy sites (two samples per quadrant). The total number of samples collected was 1760 subgingival plaque samples, and these samples were pooled into 220 samples (one sample per patient). Cheek swabs were collected using sterile plastic applicator. All samples were collected in sterile ice-chilled 5.0 mL Eppendorf tubes (Eppendorf South Pacific Pty, Ltd., Macquarie Park, NSW 2113, Australia). An amount of 0.9% sodium chloride was used in the Eppendorf tubes as preservative solution. All collected samples were stored immediately in a subzero (minus 20 °C) facility on site. A courier with specialized freezing equipment was used for the transportation of the coded samples, and all samples were sent from NK Periodontics practices in Western Australia to the Dental School, Department of Odontology, Umeå University, Sweden for subsequent processing and analysis by an experienced team of researchers.

### 2.5. Bacterial DNA Isolation

For DNA isolation from the samples, a GXT NA Extraction Kit^®^ (Hain Lifescience, GmBH, Nehren, Germany) and an Arrow automated extraction instrument (Liaison IXT, DiaSorin AB, Solna, Sweden) were used, using procedures described earlier [19].

### 2.6. Quantification PCR

The amount of total extracted DNA was quantified using a NanoDrop (Thermo Fisher Scientific, Uppsala, Sweden) instrument. For quantification of *A. actinomycetemcomitans* loads, suspensions of the reference strain HK1651 were treated as described and used for standard curves, i.e., by being serially diluted [19]. A Corbett Research Rotor-Gene 6000 Rotary Analyze instrument (QIAGEN, Valencia, CA, USA) was used for the quantification of the total concentration of *A. actinomycetemcomitans* loads in the samples, using qPCR. The cycling conditions used were according to Kirakodu method [20]. The oligonucleotide primers used were as follows: Forward (5′-CTAGGTATTGCGAAACAATTTG-3′) and Reverse (5′-CCTGAAATTAAGCTGGTAATC-3′). A load of 100 *A. actinomycetemcomitans* cells per mL of sample was set as a positive result, i.e., as a threshold, regarding presence or absence of this bacterium, in line with our recent study [18].

### 2.7. Statistical Analysis

All data collected was coded and entered into a computer using Microsoft Excel (Microsoft Corporation, 2024, Redmond, WA, USA). The collected data were analyzed using IBM SPSS Statistics software program version 29.0 (SPSS Inc., Chicago, IL, USA). The primary outcome was the presence of *A. actinomycetemcomitans* in saliva versus cheek swabs versus subgingival plaque samples using Wilcoxon test and analysis of variance. A descriptive analysis was performed, evaluating quantitative and qualitative variables, and presented in tables. Confidence level was set at 95%. The significance level used was 5%. Sample size was calculated according to previously published paper [18].

### 2.8. Ethical Considerations

The approval to conduct the current study was given by the Human Ethics, Office of Research at The University of Western Australia (2022/ET000252).

## 3. Results

*A. actinomycetemcomitans* was isolated from 28.18% (62) of the subjects (220). A total of 660 samples were obtained, 220 from unstimulated saliva, 220 cheek swabs, and 220 pooled subgingival plaque samples. One hundred and fifty-eight of the participants did not harbor the *A. actinomycetemcomitans* in the saliva, cheek swabs, and subgingival plaque area. The number of positive subjects with *A. actinomycetemcomitans* in the unstimulated saliva, cheek swabs, and pooled subgingival plaque samples were 48 (21.80%), 43 (19.50%), and 39 (17.70%), respectively. There was a statistically significant difference between the presence of *A. actinomycetemcomitans* in the unstimulated saliva samples and in the cheek, swab samples, and pooled subgingival plaque samples (*p* < 0.001). A simple linear regression evaluated the correlation of the occurrence of *A. actinomycetemcomitans* among unstimulated saliva, cheek swabs, and pooled subgingival plaque samples, which was statistically significant among the three different types of samples (*p* < 0.001).

Table 1 reveals the simultaneous and isolated detection of *A. actinomycetemcomitans* from each patient at all sampled sites. The more frequent combined detection was observed in the unstimulated saliva, cheek swabs, and pooled subgingival plaque samples. In twenty-eight *A. actinomycetemcomitans*-positive subjects, the bacterium was present in the unstimulated saliva, cheek swabs, and pooled subgingival plaque samples. In twenty-three *A. actinomycetemcomitans*-positive subjects, the bacterium was present only in the unstimulated saliva and the pooled subgingival plaque samples. In twenty of the *A. actinomycetemcomitans*-positive subjects, the bacterium was present only in the unstimulated saliva samples and the cheek swabs. Finally, in seventeen of the *A. actinomycetemcomitans*-positive subjects, the bacterium was present in the cheek swabs and the pooled subgingival plaque samples. In the *A. actinomycetemcomitans*-positive subjects, the isolated detection of the bacterium was not common. In ten *A. actinomycetemcomitans*-positive subjects, the bacterium was isolated from cheek swabs. In nine *A. actinomycetemcomitans*-positive subjects, the bacterium was isolated from the unstimulated saliva samples. Finally, in only three of the *A. actinomycetemcomitans*-positive subjects, the bacterium was isolated from the pooled subgingival plaque samples alone.

Table 2 shows the *A. actinomycetemcomitans*-positive patients and their characteristics. The overall mean DNA concentration of *A. actinomycetemcomitans* in all the sixty-two positive subjects from all intra-oral sampled sites (unstimulated saliva, cheek swabs, and pooled subgingival plaque) was 633,160 ng/μL (SD ± 48,288.01). On the other hand, the overall mean DNA concentration of *A. actinomycetemcomitans* in all the one hundred and fifty-eight negative subjects from all intra-oral sampled sites was 5.23 ng/μL (SD ± 14.88). The mean DNA concentration of *A. actinomycetemcomitans* in the unstimulated saliva samples was 491,799 ng/μL (SD ± 30,335.56) in *A. actinomycetemcomitans*-positive patients (*n* = 48) compared to 0.57 ng/μL (SD ± 5.97) in the *A. actinomycetemcomitans*-negative patients (*n* = 172). The mean DNA concentration of *A. actinomycetemcomitans* in cheek swabs was 851.47 ng/μL (SD ± 4541.72) in *A. actinomycetemcomitans*-positive patients (*n* = 43) compared to 10.06 ng/μL (SD ± 20.16) in the *A. actinomycetemcomitans*-negative patients (*n* = 177). The mean DNA concentration of *A. actinomycetemcomitans* in pooled subgingival plaque samples was 1,322,533 ng/μL (SD ± 77,431.91) in *A. actinomycetemcomitans*-positive patients (*n* = 39) compared to 5.08 ng/μL (SD ± 13.37) in the *A. actinomycetemcomitans*-negative patients (*n* = 181).

## 4. Discussion

*A. actinomycetemcomitans* is a key periodontal pathogen associated with periodontitis in terms of diagnosis, vaccination, and treatment. In the present study, the prevalence of this bacterium was observed in 28.18% of the oral cavities of participants diagnosed with an advanced form of periodontitis; this was higher than in our earlier study that looked at the subgingival population of *A. actinomycetemcomitans* only [18]. The population included in this study revealed a statistically significant correlation between the severe form of periodontitis (stage IV, grade C) and younger age groups, family history of periodontitis, lack of dental flossing, irregular dental care, and those who lost their teeth due to periodontitis [21]. The prevalence of *A. actinomycetemcomitans* in our study is still below that in the African and Asian studies. However, it is still higher than in the European studies; these results must be interpreted with great caution, as we used samples from three different oral sources compared to other studies that used one or two sources [7,22,23,24,25,26,27,28,29,30]. Regarding the intraoral site of detection of *A. actinomycetemcomitans*, the unstimulated saliva had the highest frequency of detection followed by cheek swabs and pooled subgingival plaque samples. Some of the cheek swabs did not have a bacterium concentration above the detection threshold (29 samples). In one Brazilian study that included 66 adults with generalized chronic periodontitis, samples from unstimulated saliva, tongue, cheek, and subgingival plaque were collected [22]. The overall prevalence of *A. actionomycetemcomitans* in the 66 subjects was 86.36%, which is much higher than the present study. The subgingival samples were collected from the deepest two periodontal pockets in each quadrant (8 samples/patient). The presence of *A. actionomycetemcomitans* was established using bacterial culture. The bacterium was isolated from 63.63% of subgingival plaque samples, 56.06% of saliva samples, and 45.45% of samples from mucous surfaces; these findings are in agreement with other studies [31]. In another study using culture technique to analyze the intraoral distribution of *A. actinomycetemcomitans* in 17 young adults with mild periodontitis, the bacterium was isolated from 62% of the cheek samples, 59% of salivary samples, and 41% of samples collected from palatal tonsils [32]. Despite the large number of subgingival plaque samples (1760 samples) isolated from the 220 patients in the present study, only 39 of them were positive (17.70%); this was lower than the cheek swabs (*n* = 43 subjects–19.50%). However, pooled subgingival samples had the highest concentration of *A. actinomycetemcomitans* compared to the unstimulated saliva and cheek swabs. In one study, higher levels of *A. actinomycetemcomitans* were found in both saliva and plaque samples from the periodontitis group in comparison to the healthy subjects using the quantitative PCR technique [33]. In this study, the periodontitis group harbored a higher prevalence of *A. actinomycetemcomitans* both in saliva and diseased sites, whereas they were completely absent at the healthy sites and in samples from non-periodontitis subjects [33]. These findings are consistent with those of other studies [34]. In a study by Claesson and coauthors assessing 3459 subgingival plaque samples isolated from 1445 patients, 30% of the sampled patients were positive for *A. actinomycetemcomitans,* with the younger age group having a higher portion of this bacterium compared to the older age group [35].

Biological unstimulated saliva sampling might be a promising method of microbial diagnosis in periodontitis, as this fluid can be easily, repetitively, and non-invasively collected very quickly in a chairside manner. However, several studies have shown that the bacterial composition of saliva differs significantly from that of dental biofilm in the gingival crevicular fluid [36]. A previous study also revealed a difference in the microbiota community structure between supragingival plaque and saliva, and the compositional stability of salivary microbiota against a supragingival microbiota shift [37]. Due to its easy, quick, and non-invasive sampling, salivary microbiota quantification has been successfully used in several studies and has been proposed as a diagnostic biomarker for periodontitis either in periodontally healthy subjects or in subjects with periodontitis subjects [38,39,40]. However, the number of studies that have used the quantitative PCR technique to detect and quantify periodontal pathogens in distinct study groups is limited. In our study, *A. actinomycetemcomitans* detection in unstimulated saliva was very high using the quantitative real-time PCR technique. The limitations of the present study were the lack of control groups (healthy periodontia), the inability to detect a cause–effect relationship, and the inability to guarantee results representation due to the snapshot nature. All patients were referred to a periodontal practice, so most of them were periodontally unstable.

## 5. Conclusions

The results of this cross-sectional study in subjects with advanced periodontitis suggest that saliva is representative of pooled subgingival plaque and cheek swab samples, and its use is appropriate in the detection of *A. actinomycetemcomitans*. However, these results must be considered with caution and further analysis must be carried out.

## Figures and Tables

**Table 1:** Frequency of *A. actinomycetemcomitans* detection.

	Sample Sites	Number of Patients
Simultaneous detection	Saliva, check swabs, and pooled subgingival plaque	28
	Saliva and pooled subgingival plaque	23
	Saliva and check swabs plaque	20
	Check swabs, and pooled subgingival plaque	17
Isolated detection	Cheek swabs	10
	Saliva	9
	Pooled subgingival plaque	3

**Table 2:** *A. actinomycetemcomitans*-positive patients and their characteristics.

Patient/Variables	Sex	Age in Years	Stage/Grade	*Aa* Saliva ng/μL	*Aa* Cheek Swabs ng/μL	*Aa* Plaque ng/μL
Pt 1	F	70	III/B	72,055	-	848,041
Pt 2	F	32	III/C	-	119	-
Pt 3	M	30	III/B	-	1956.5	-
Pt 4	M	40	IV/B	-	511	-
Pt 5	F	63	III/B	-	219	-
Pt 6	M	48	IV/C	19,950.5	2679	22,379
Pt 7	F	56	III/B	1075	161	-
Pt 8	F	48	III/B	606.50	-	10,401
Pt 9	F	40	IV/C	1470	638.5	1719
Pt 10	M	49	III/B	8530	-	1680
Pt 11	M	56	III/B	14,290	165.5	1448
Pt 12	M	54	III/B	2500	-	303
Pt 13	M	35	III/B	3810	-	-
Pt 14	M	68	IV/C	9820	570.5	365,681
Pt 15	F	51	II/B	5124	-	-
Pt 16	M	34	IV/C	8685	2299.5	851
Pt 17	M	57	III/B	6865	-	-
Pt 18	F	56	III/B	-	-	153
Pt 19	F	65	IV/C	13,910	247.5	10,651
Pt 20	M	57	III/B	4115	-	-
Pt 21	M	39	III/B	2415	-	577
Pt 22	F	40	III/B	26,620	1219.5	153
Pt 23	M	57	III/B	2065	-	-
Pt 24	M	33	III/C	1710	-	-
Pt 25	M	64	III/B	1050	-	-
P 26	F	71	IV/C	1325	-	-
Pt 27	F	62	III/B	76,820	2716	381,842
Pt 28	F	36	III/B	2195	559	4624
Pt 29	M	40	III/B	-	-	340
Pt 30	M	57	III/B	1255	204	56,334
Pt 31	F	47	III/B	411,735	604.5	46,227
Pt 32	M	40	III/B	54,790	153	184,822
Pt 33	M	75	III/B	-	-	132
Pt 34	M	56	III/B	1510	-	479,910
Pt 35	M	52	III/B	3360	22,932	5163
Pt 36	F	40	IV/C	-	105	-
Pt 37	F	52	III/B	120,725	23,264.5	-
Pt 38	F	60	IV/C	33,910	25,995	254,437
Pt 39	M	76	III/B	-	105	-
Pt 40	F	69	III/B	-	133.50	-
Pt 41	M	62	III/B	-	945.5	-
Pt 42	F	40	III/B	4965	43,559	81,934
Pt 43	F	32	III/B	54,440	139,50	-
Pt 44	M	49	III/B	13,495	-	-
Pt 45	F	49	IV/C	46,010	503.5	-
Pt 46	M	68	III/B	-	353	3598
Pt 47	M	37	IV/C	-	106.5	-
Pt 48	M	33	III/B	4095	30,455.5	101,611
Pt 49	M	63	III/B	-	114.5	-
Pt 50	M	66	III/B	1525	-	138.50
Pt 51	M	40	IV/C	564	237	262
Pt 52	F	38	II/B	8845	3782	3978
Pt 53	M	38	III/B	12,377	5135	5432
Pt 54	M	33	IV/C	5567	4822	19,792
Pt 55	F	36	III/B	4133	1103	1281
Pt 56	M	40	III/B	1298	856	1234
Pt 57	F	39	III/B	1593	1104	3789
Pt 58	F	39	III/B	2267	1409	1817
Pt 59	F	32	IV/C	1621	1381	627
Pt 60	F	36	IV/C	656	263	449
Pt 61	M	38	III/B	3250	3063	5343
Pt 62	F	32	III/B	996	429	419

## Data Availability

The data supporting this study’s findings are available by emailing the corresponding authors.
